# Supplementary material for: Activation of plant immunity through conversion of a helper NLR homodimer into a resistosome
Source: PLoS Biol. 2024 Oct 18;22(10):e3002868. doi: 10.1371/journal.pbio.3002868 (PMC11524475; doi:10.1371/journal.pbio.3002868)
Supplement: S1 Table — (DOCX) [file pbio.3002868.s013.docx]

| Data collection and processing | Resting state NRC2  PDB: 8RFH - EMDB: 19121 |
| --- | --- |
| Microscope | Titan Krios |
| Voltage(KeV) | 300 |
| Detector | Gatan K3 |
| Magnification | 105,000 |
| Calibrated pixel size (Å) | 0.828 |
| Exposure time (s) | 2.6 |
| Frames per exposure (e^-^/Å^2^) | 50 |
| Total electron exposure | 50 |
| Automation software | EPU |
| Defocus range (mM) | 1.5 to 2.7 |
| Number of micrographs used | 6135 |
| Total refined particles (no.) | 229,347 |
| Symmetry imposed | C2 |
| Map sharpening B-factor (Å^2^) | -100 |
| Unmasked Resolution at 0.5/0.143 FSC (Å) | 4.1/4.5 |
| Masked resolution at 0.143/0.5 FSC (Å) | 3.9/4.2 |
| Model refinement and validation statistics |  |
| PDB composition | 2 Protein chains |
| Amino acids | 1469 |
| Ligand | ADP |
| RMSD bonds (Å) | 0.002 |
| RMSD angles (°) | 0.773 |
| B-factor | 176 |
| Ramachandran  Favoured (%)  Allowed (%)  Outliers (%) | 97.51  2.49  0.0 |
| Rotamer outliers (%) | 0.30 |
| Clash score | 15.89 |
| C-beta outliers (%) | 0.0 |
| CaBLAM outliers (%) | 0.97 |
| CC (mask) | 0.75 |
| CC (volume) | 0.732 |
| Molprobity score | 1.8 |
| EMRinger | 0.87 |

**S1 Table: Cryo-EM data collection, refinement, and validation statistics.**
