# Supplementary material for: Activation of plant immunity through conversion of a helper NLR homodimer into a resistosome
Source: PLoS Biol. 2024 Oct 18;22(10):e3002868. doi: 10.1371/journal.pbio.3002868 (PMC11524475; doi:10.1371/journal.pbio.3002868)
Supplement: S2 Table — (DOCX) [file pbio.3002868.s014.docx]

**S2 Table: Interface residues at 6 Å cut-off distance and their definition as amino acid stretches.**

| **Interfaces 1 and 3** | NB domain region: Y217,R220,H238,D239,M240,C241,E242,E243,D244. D270 and R274.  LRR domain region:  R513,K512,G511,S536,K563,P559,F509,Y508,E506,K534,T560,S532, P535,T533 |
| --- | --- |
| **Interface 2** | LRR region:  Q507,S532,E529, L528,P554,M553,E552,K549 |
| **Amino acid stretches involved in interfaces 1 and 3** | - Stretch1a:217-220 - Strech1b: 238-244 - Strech1c: 270-274 - Strech1d: 506-513 - Stretch 1e: 533-536 - Stretch 1f: 559-563 |
| **Amino acid stretches involved in interface 2** | - Stretch2a:  528-532 - Stretch 2b: 549-554 |
