# Supplementary material for: Activation of plant immunity through conversion of a helper NLR homodimer into a resistosome
Source: PLoS Biol. 2024 Oct 18;22(10):e3002868. doi: 10.1371/journal.pbio.3002868 (PMC11524475; doi:10.1371/journal.pbio.3002868)
Supplement: S1 Raw Images — (PDF) [file pbio.3002868.s029.pdf]

Uncropped blots accompanying Figure 1A

Western blots with MW marker superimposed

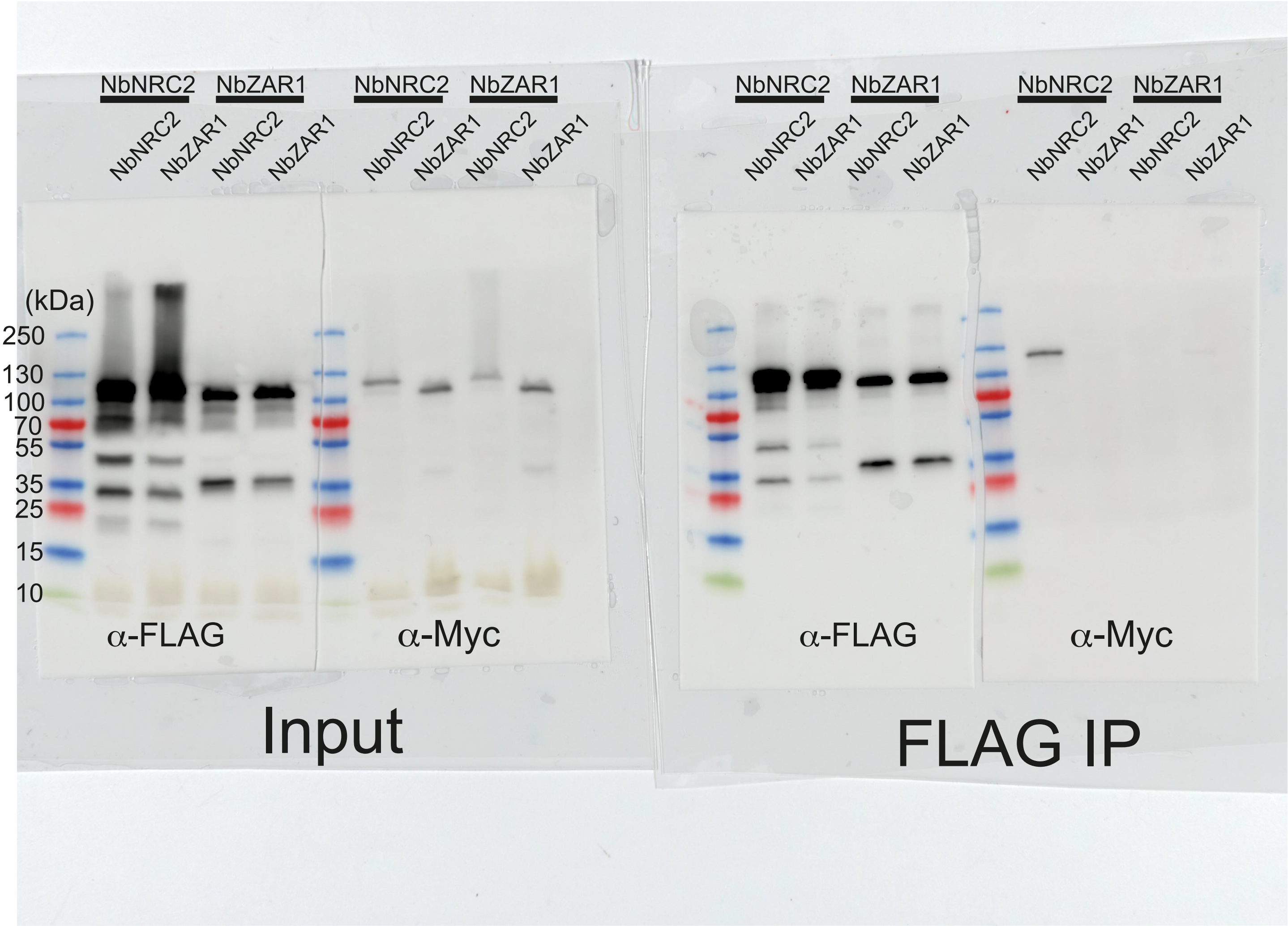

Loading control done by Ponceau Stain

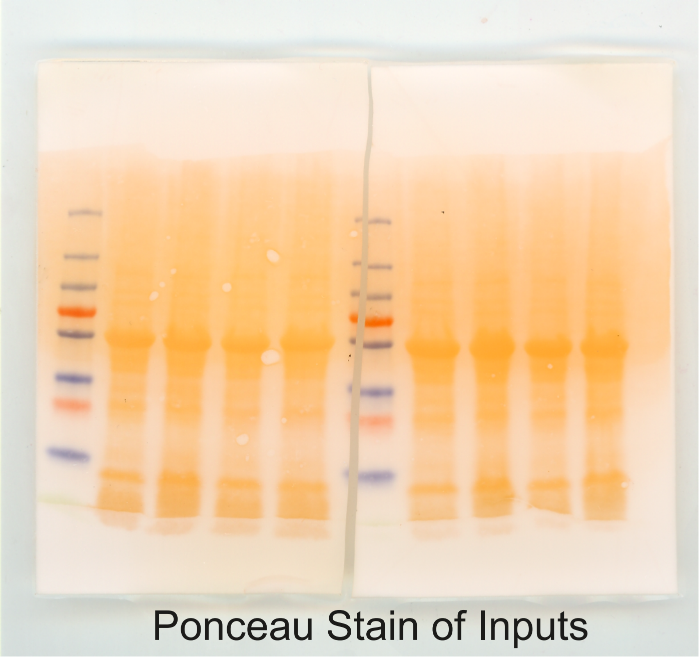

Uncropped blots accompanying Figure 2B

Western blots with MW marker superimposed

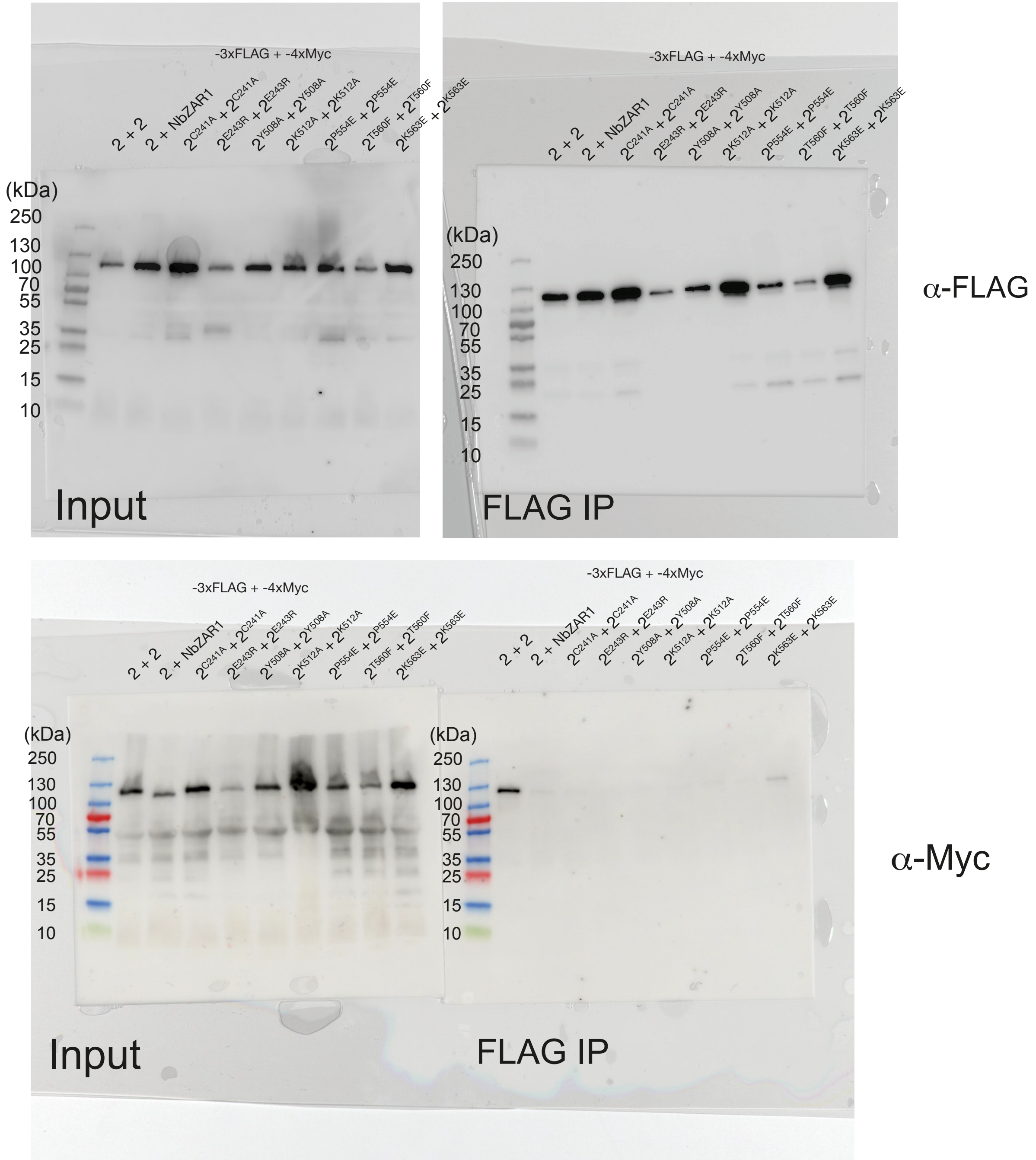

Loading control done by Ponceau Stain

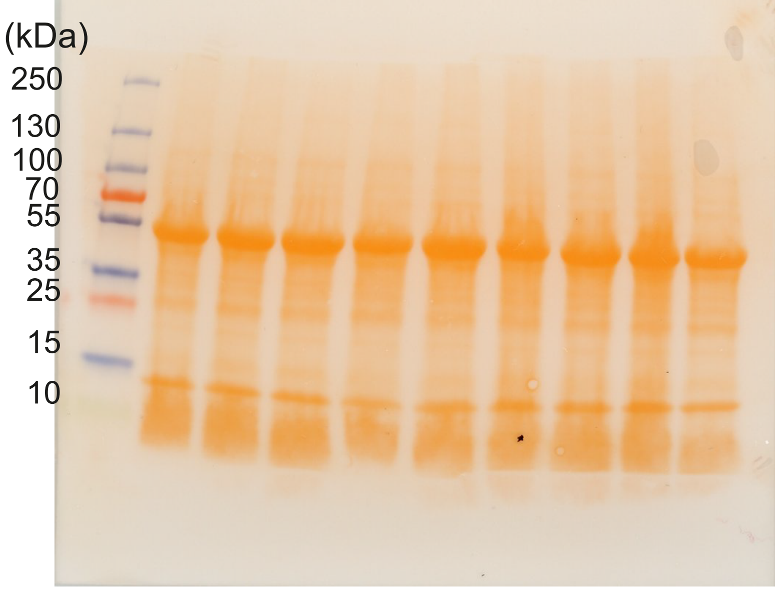

Uncropped blot accompanying Figure 4A superimposed with brightfield images to visualize molecular weight ladder

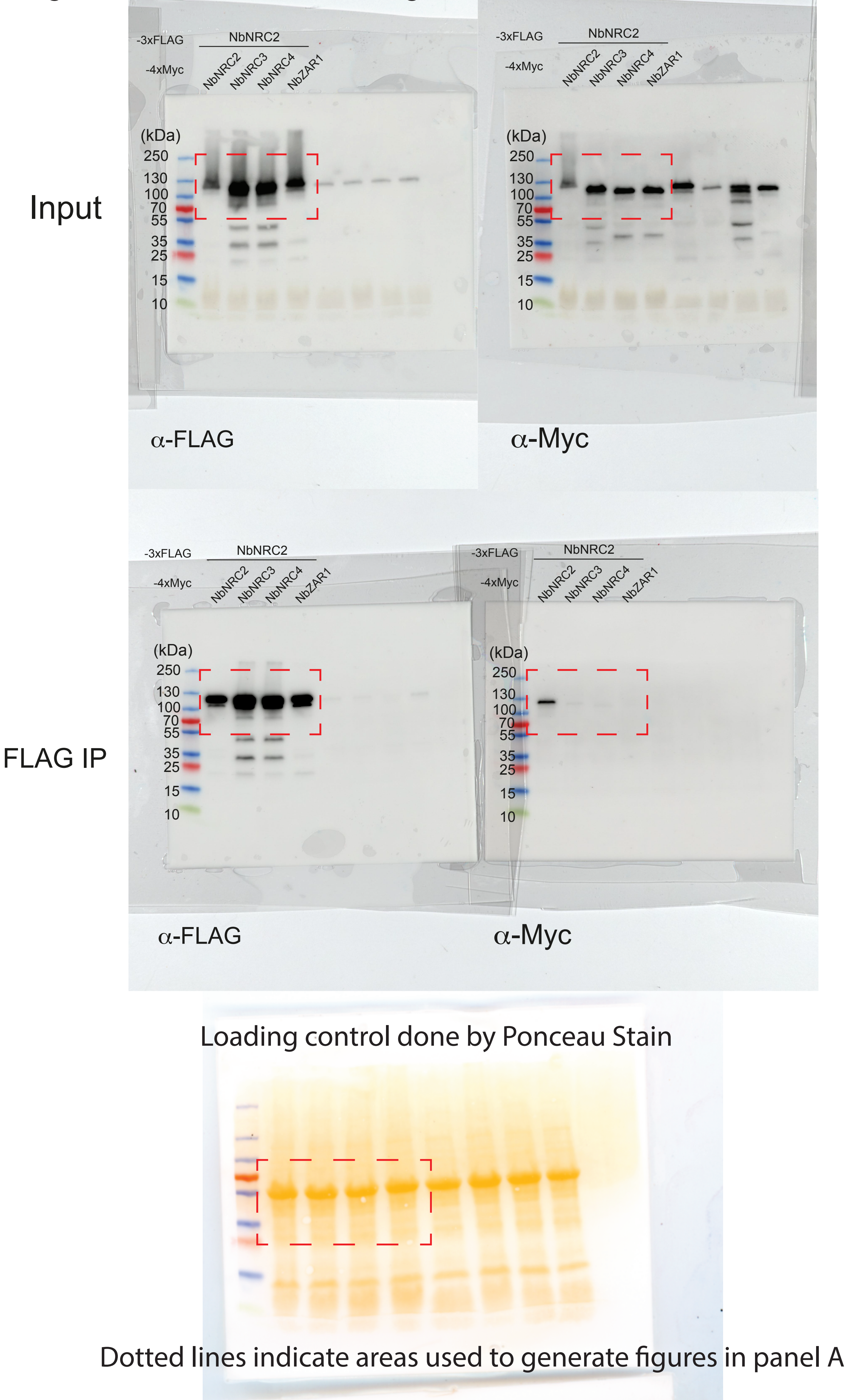

Dotted lines indicate areas used to generate figures in panel A

Uncropped blot accompanying Figure 5C superimposed with brightfield images to visualize molecular weight ladder

Input

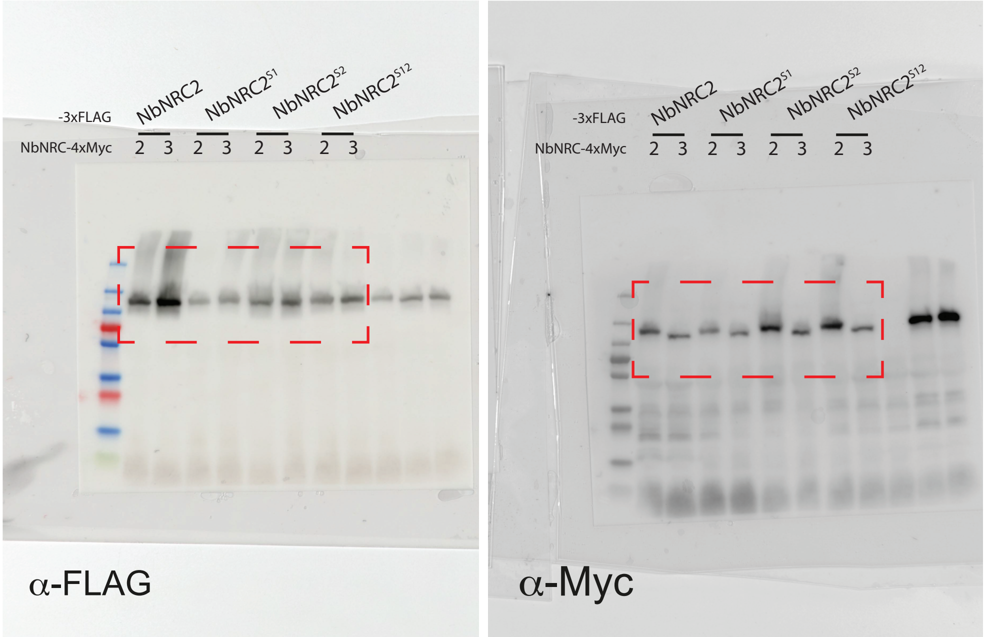

FLAG IP

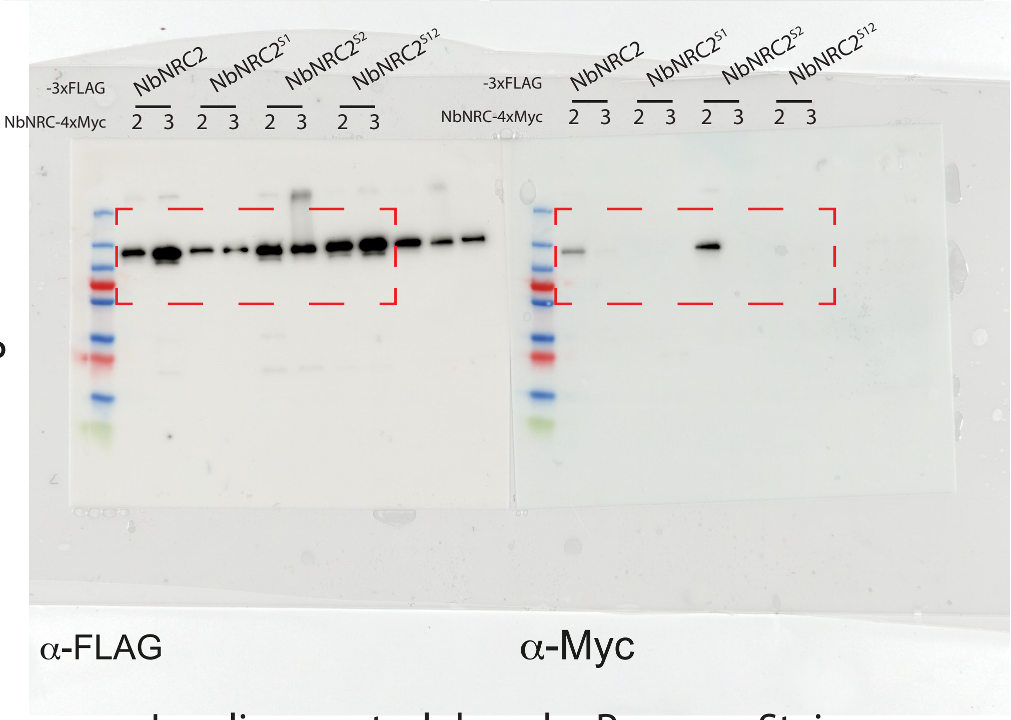

Loading control done by Ponceau Stain

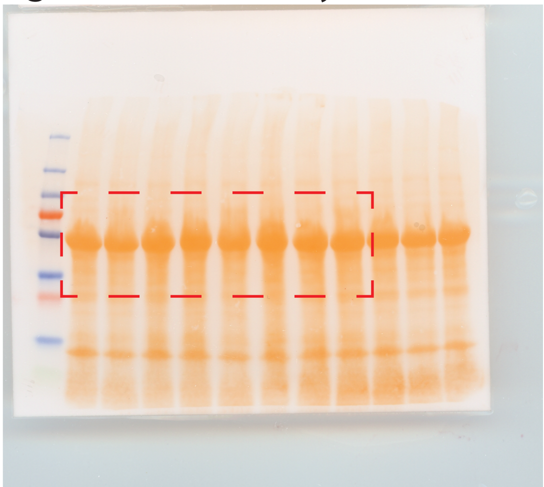

Dotted lines indicate areas used to generate figures in panel C

Uncropped gel scan accompanying Figure S1

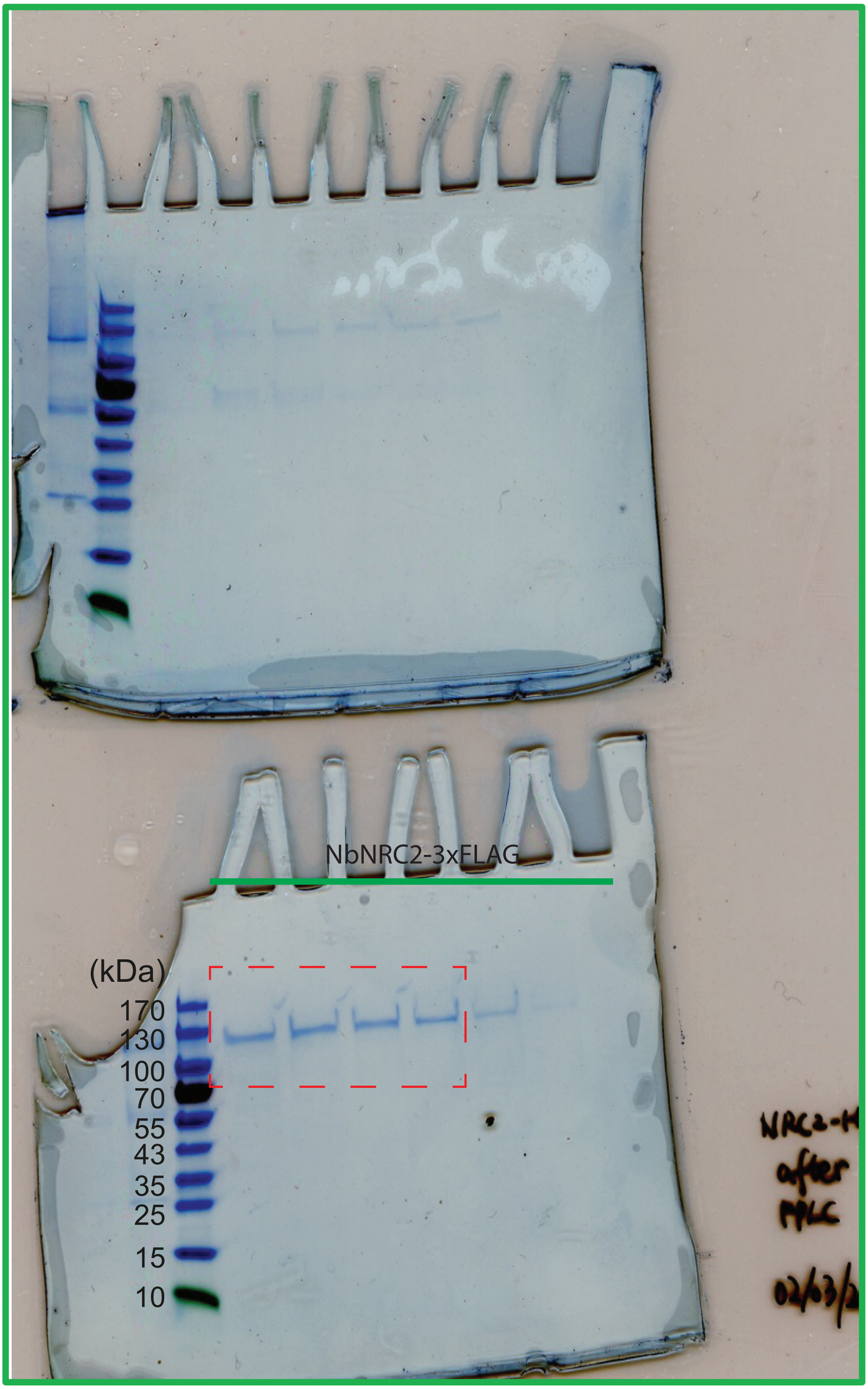

Dotted lines indicate areas used to generate Figure in panel S1

Uncropped blots accompanying Figure S5 superimposed with brighfield image to visualize molecular weight marker

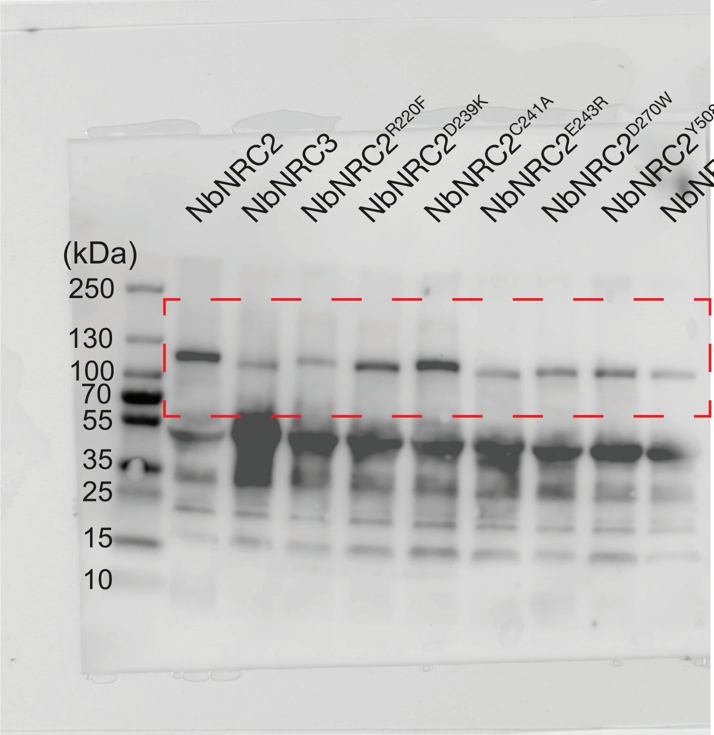

α-Myc

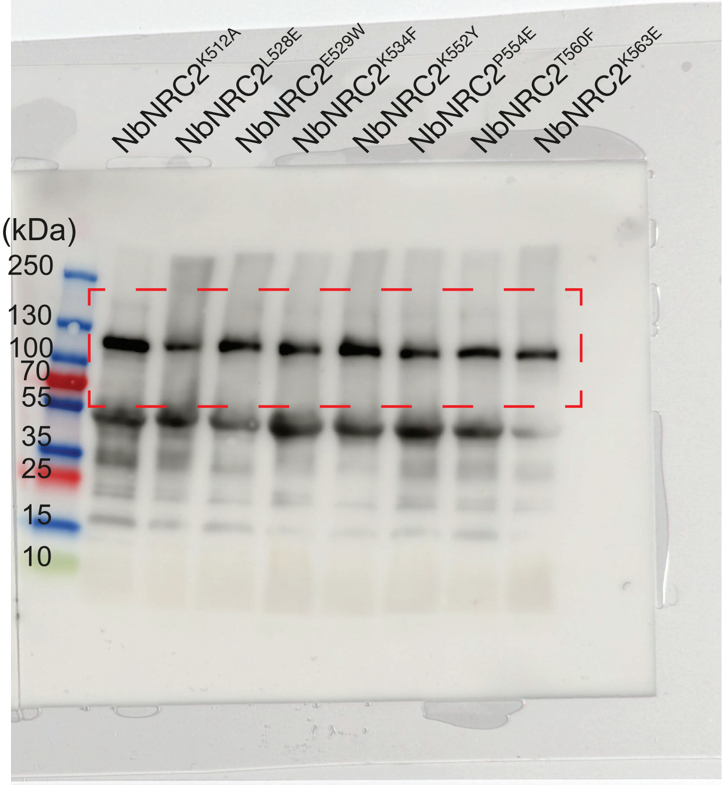

α-Myc

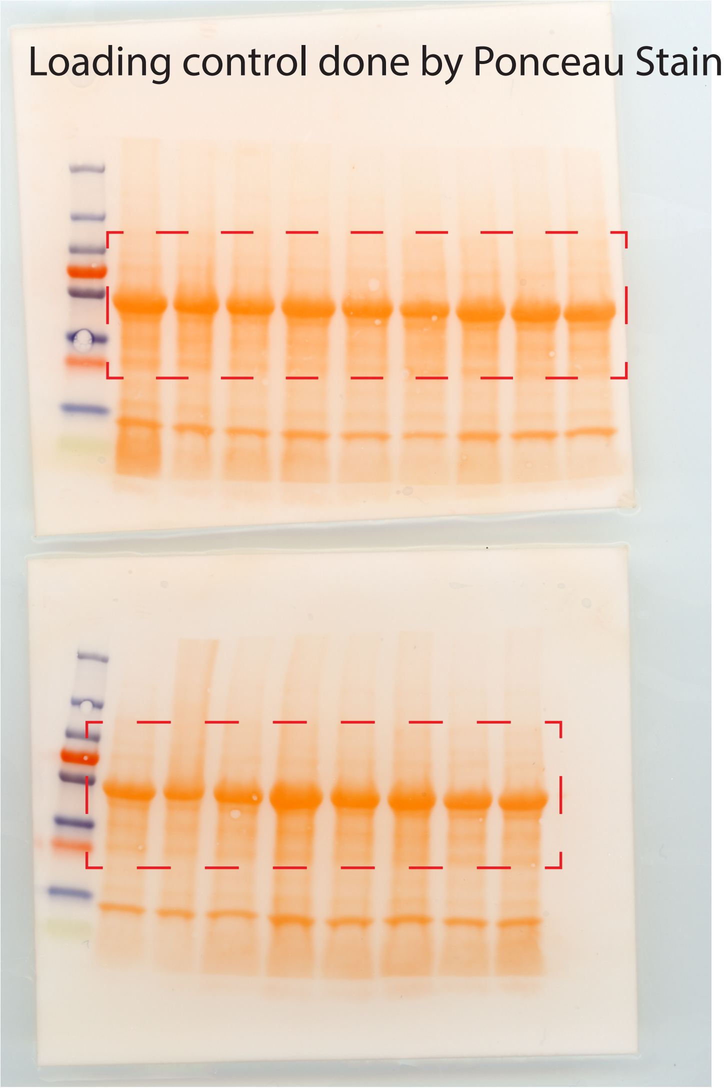

Dotted lines indicate areas used to generate Figure in panel S1

Uncropped blot accompanying Figure S8 A superimposed with brightfield images to visualize molecular weight ladder

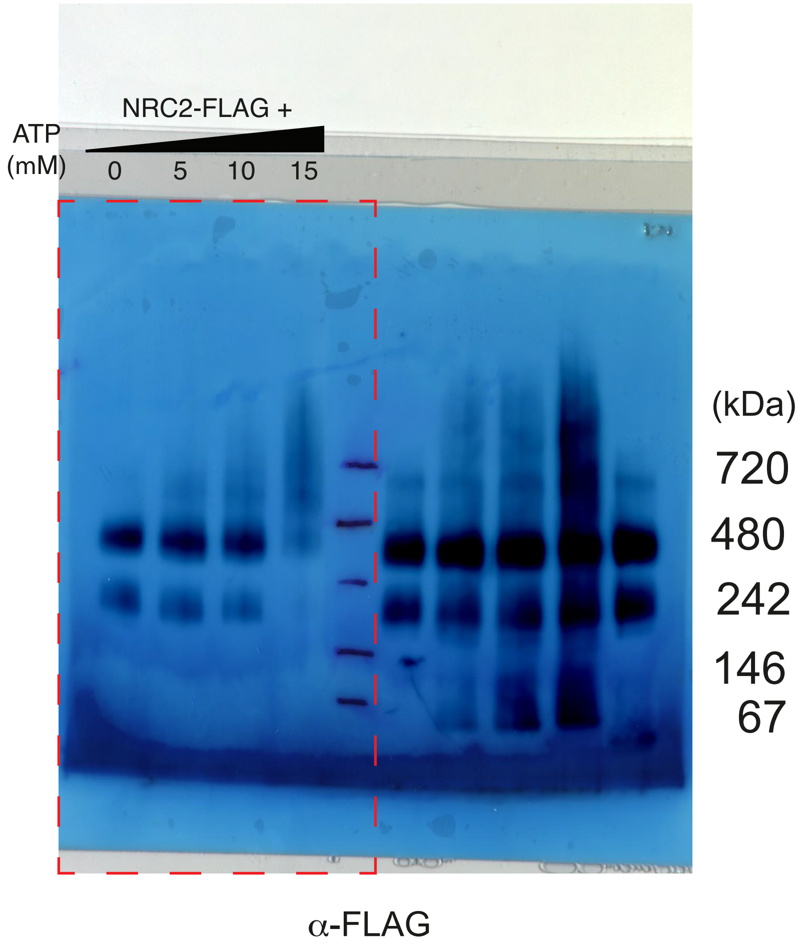

Dotted lines indicate area used to generate figure in panel A

Uncropped blots accompanying Figure S8 B superimposed with brightfield images to visualize molecular weight ladder

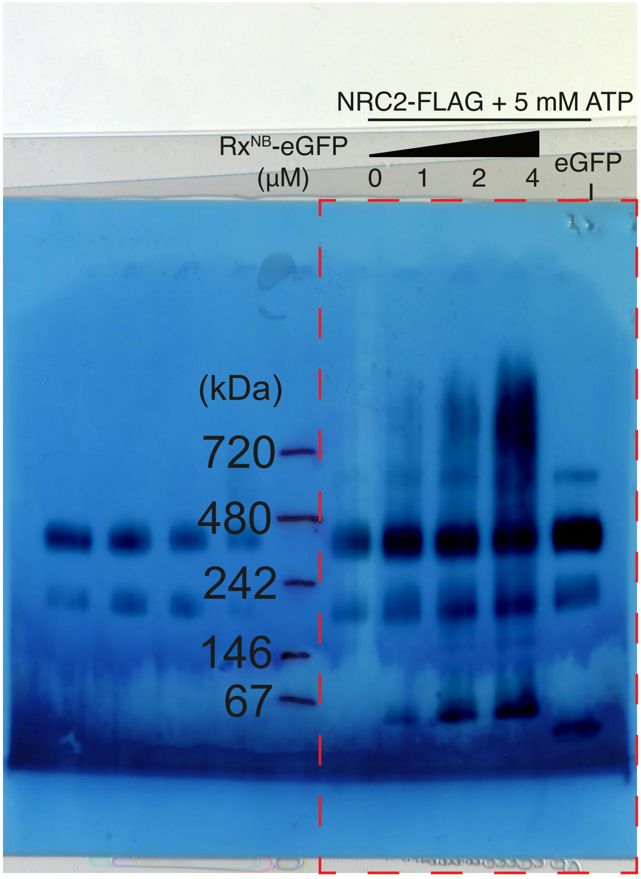

α-FLAG

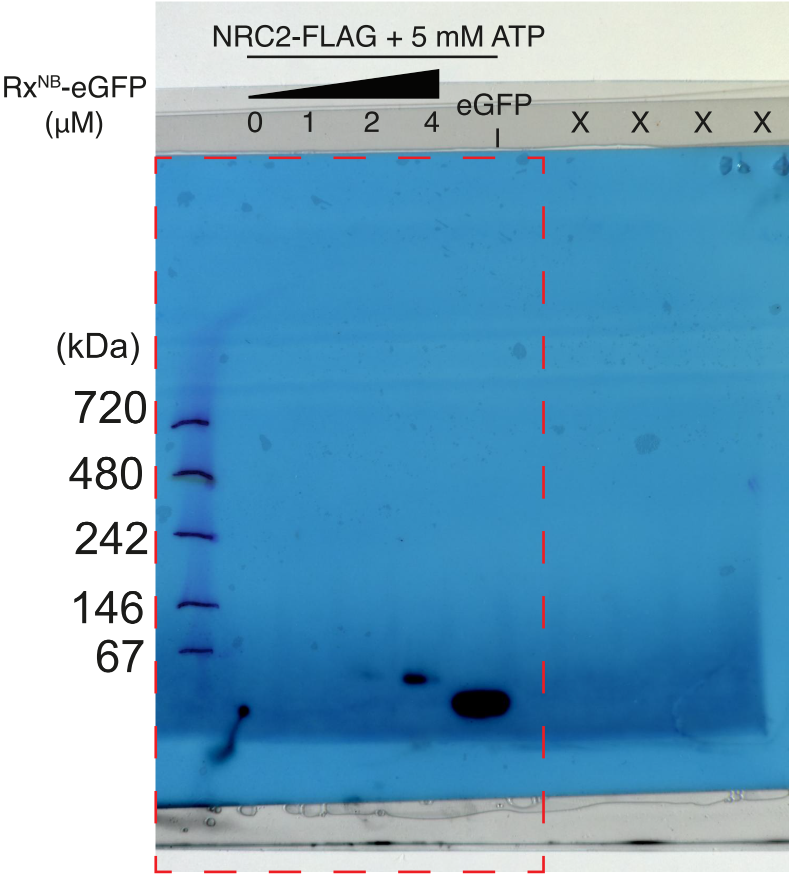

α-GFP

Dotted lines indicate areas used to generate figures in panel B

Uncropped blot accompanying Figure S8 C superimposed with brightfield images to visualize molecular weight ladder

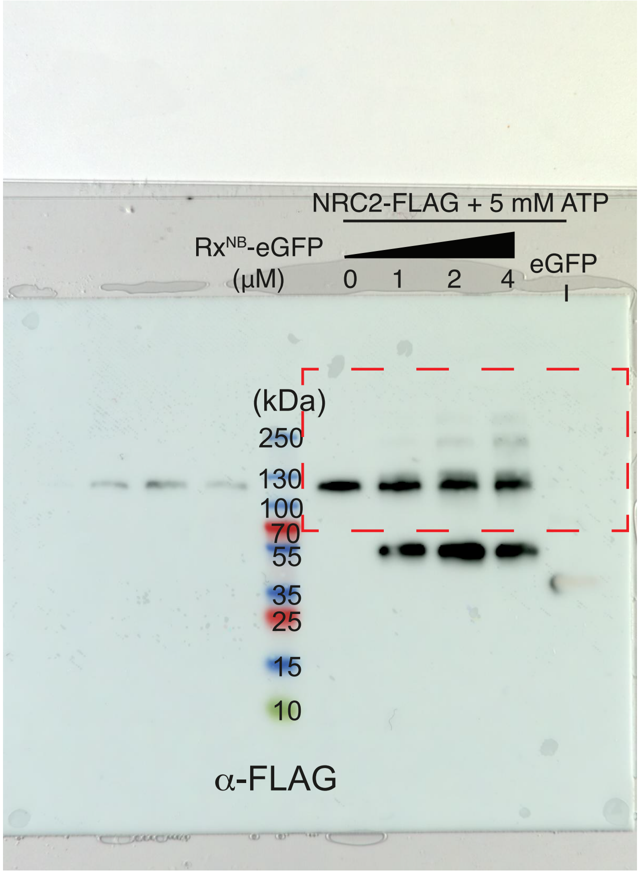

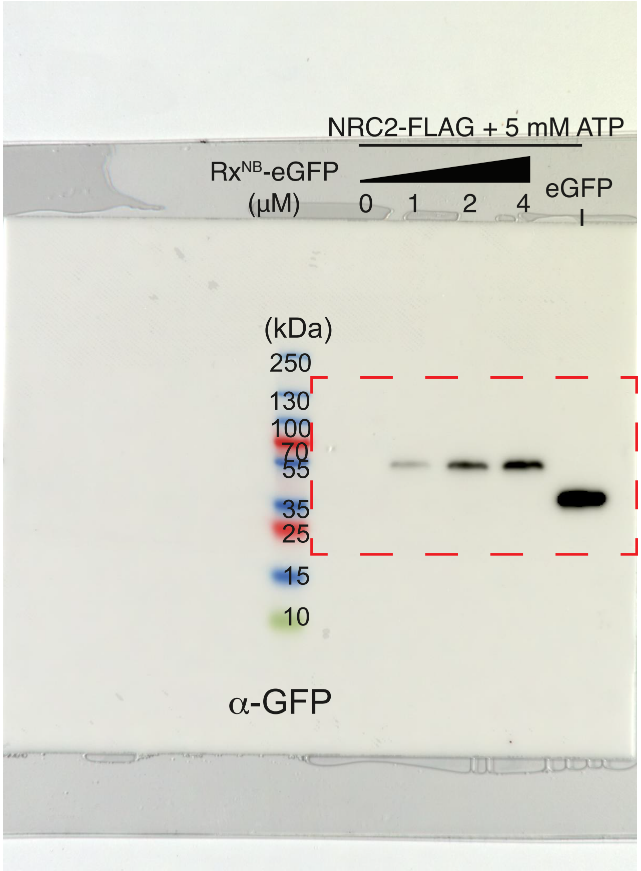

Dotted lines indicate areas used to generate figures in panel C
