# Supplementary material for: Activation of plant immunity through conversion of a helper NLR homodimer into a resistosome
Source: PLoS Biol. 2024 Oct 18;22(10):e3002868. doi: 10.1371/journal.pbio.3002868 (PMC11524475; doi:10.1371/journal.pbio.3002868)
Supplement: Attachment — Submitted filename: 240804_SelvarajResponseToReviewers.docx [file pbio.3002868.s030.docx]

Dear Dr. Vázquez Hernández

Thank you very much for your evaluation of our manuscript "Activation of plant immunity through conversion of a helper NLR homodimer into a resistosome" for consideration as a research article at PLoS Biology.

We appreciate the overall positive feedback of the independent reviewers and have made the suggested changes.

We now include new experiments to comprehensively address the reviewer comments. Please see the point-by-point 'Response to Reviewers' below.

We hope that we have addressed many of the issues that were raised, and we look forward to your response.

Best regards,

Sophien Kamoun

Group Leader and Professor

Mauricio P. Contreras

PostDoctoral Scientist

Reviewer #1: 

In this manuscript (Short Reports), Selvaraj and co-workers report the cryoEM structure of the homodimeric NbNRC2, a helper NLR, at 3.9 A resolution. Incubation of NbNRC2 with high concentrations of ATP or the nucleotide-binding (NB) domain of the disease resistance protein Rx triggers NbNRC2 oligomerization. The authors show that the dimerization of the NbNRC2 is highly specific, as NbNRC2 does not pulldown other paralogs, such as NbNRC3, NbNRC4, or NbZAR1.  This specificity arises from the distinct pattern of interfacial residues in individual NRC clades.  Overall, this is a well done study that provides important insights into the structure and activation of NbNRC2.  However, there are a few issues that should be addressed before publication.

1. The authors have discovered patterns of the interfacial residues that dictate the specificity of NbNRC2 and paralogs.  Yet, no effort is made to validate this observation.  Can the authors swap interfacial residues to alter the binding partner?

We thank the reviewer for this suggestion. We have generated chimeric NbNRC2-NbNRC3 proteins where amino acid stretches at the homodimerization interface were swapped. These variants were tested in CoIP assays, which revealed that swapping interfacial residues of NbNRC3 Interface 1 (NB domain LRR interface) into NbNRC2 resulted in loss of association with NbNRC2, suggesting that these residues indeed contribute to homodimerization. Interestingly, swapping residues at interface 2 (LRR-LRR interface) did not abolish homodimerization, suggesting that interface 1 is more critical for homodimerization than interface 2. None of the swaps tested gained association with NbNRC3, suggesting that there are potentially more residues driving incompatibility. We have included all of these data in a new figure.

2. The discussion and experimental analysis of the Rx-triggered oligomerization of NbNRCs is confusing. Is it truly an oligomer or a hexamer?  What is the experimental evidence?  The native gel shift result in Figure 2 does not define the oligomeric state.  Can the authors use crosslinking agents to define the oligomeric state?

We thank the reviewer for this comment. We previously addressed the issue of oligomerization in two previous publications, Contreras et al. (2022) EMBOJ, Contreras et al. (2023) Sci. Adv. and Ahn et al. (2023) EMBOJ. In these papers, we used non-denaturing BN-PAGE assays to show that sensor NLR activation with pathogen effectors or upon infection by pathogens leads to oligomerization of helper NLRs. Since submission to PLoS, we have also addressed the precise nature of the stoichiometry of the Rx-activated NbNRC2 oligomer in our recent preprint, where we obtained the cryo-EM structure of this oligomer (see Madhuprakash et al. 2024, on bioRxiv https://www.biorxiv.org/content/10.1101/2024.06.18.599586v1). This confirmed that indeed, Rx activation with PVX CP leads to the assembly of NbNRC2 homo-hexamers. We have now included a section covering the recently characterized NbNRC2 homo-hexamers in our manuscript.

------------------------------------


Reviewer #2: 

Selvaraj and colleagues present their work determining the cryo-EM structure of NRC2 dimer purified from N. benthamiana as well as showing that three regions between NBD and LRR are involved in homo-dimerization. Given the genetic redundancy of NRC helper NLRs, the authors tested the hypothesis of Rx-mediated NRC2 resistosome formation using BN-PAGE on its own and confirmed that it is a likely scenario that is ATP-concentration dependent. The authors further demonstrated that NRC2 NLR prefers to form a homomeric complex in plants using Co-IP experiment even in the presence of the other NRC helper paralogs in its companion, and do not localize with the other paralog NLR4 in the plant cell when transiently expressed. The evolutionary analyses of the NRC members revealed that the residues at the dimeric interface underwent for diversification, implying that these residues might have played a role in insulating respective helper nodes from the others.

While the authors presented an interesting model of dimeric helper NRC2 as a major form existing in the resting state, which distinguishes itself from the first resistosome Zar1 model, the breadth of presented data appears to remain premature to strongly support the conclusion.
1.    The authors reported the resolution as 3.9Å. However, there are several issues regarding the cryo-EM structure. The FSC curve plunges within a narrow resolution range indicating that the resolution may be overestimated, may be due to too tight mask or others. In addition, the maps shown in Supple Fig. 2 and Supple Fig. 3 do not align with a 3.9 Å resolution. These indicate that the resolution of the cryo-EM structure is highly overestimated. Furthermore, the authors refined the atomic model using the PHENEX real space refinement. As the real space refinement replies on the quality of the map, the current map quality would not allow to perform real space refinement.

We thank the reviewer for these comments. The structure we determined in graphene oxide backing has preferred orientation, with particles orienting mostly with their long axis perpendicular to the view, imparting some level of anisotropic views. The particle is small (170kDa) with low symmetry. We now describe our resolution as ‘ranging from 3.9 to 4.5Å as per local resolution distribution in protein surface (supplementary figure workflow) in the main text. Also, we are including close up views of areas where we begin to see side-chains up to C-beta atoms clearly, along with resolving features for individual beta-strands in the post-processed map at selected places where they are clearly visible. We also have now included details on mask creation, how soft it covers the map, and a close-up view of mask covering the 3D-volume used for post-processing. Additionally, we have now included a Phenix autosharpened map of postprocessed map in the supplementary figure, showing structural features further clearly. This auto-sharpening module in Phenix is an optional tool to visualize map features by blurring/sharpening (but not for refinement). Throughout our analysis, our structure and map are interpreted in terms of domain organization and their relative orientation without entering into side chain interactions as the map does not allows it. For this reason, we used a relaxed cut-off of 6Å while selecting residues at the interface, to include most of the rotomer conformation of residues at the interface in this resolution. Our pixel size is 0.868Å. The reconstruction was carried out without binning as the particle size can be fitted in a reasonably small box (256 pixels) and the number of images are not very high. Since the Nyquist limit is far from our resolution, the plot looks steeper in our case. In their two Acta D papers, on describing ‘phenix_autorefine’ software for realspace cryo-EM refinement, the authors/developers have used this software to refine protein structures upto 6Å resolution or better (Acta Cryst. (2018). D74, 531–544 (please see in section 3.2.2 in this paper); Acta Cryst. (2021). D77, 48–61;). We performed phenix-realspace refinement the fitted model to optimize the geometry and relieve short contacts in the protein chains. So we used this software for our molecule after rigid-body placement of individual domains into the map and manual adjustments.

2.    Despite the resolution issue in the cryo-EM structure, the structure delineates the interaction between two NRC2 molecules, which probably enables the authors to map the interaction residues in the dimerization surface. Therefore, it is surprising that the author did not attempt to validate the interface in-vitro and in-vivo. The author should design mutants to disrupt the interface and biochemically shows the mutants show a monomer. From the evolutionary analyses, the authors seem to already have identified relevant unique residues found in NRC2 and NRC3. It will be necessary to see at least if these evolutionary wired residues have impact on functional outcomes, e.g. strengthening dimer interfaces, helper function and specificity, when tested in each of the NRC molecules.

Thank you for this comment. We have now generated an array of point mutants at the homodimerization interface. This mutagenesis analysis revealed that mutations at the homodimerization interface in many cases lead to auto-activity, suggesting that this interface is contributing to helper NLR autoinhibition. We also tested homodimerization interface mutants in CoIP assays, and found that they are also affected in self-association. All in all, our data seem to support a model in which homodimerization contributes to NRC autoinhibition.

3.    More importantly, biological implication of the dimeric interface shall be further corroborated. The authors speculated that multiple residues at the dimeric interface would play a role in insulating NRC2 signalling from the other helper NLRs action. Once the authors obtain which residues are critical in maintaining the dimeric interface of NRC2 homodimer (related to #3), the author should be able to show that how the stable dimeric formation contribute to fine-tune rather initially thought redundancy helper network or to facilitate NRC2-specific immune responses. Without in-vitro and in-vivo validation, this work is premature to be considered for a publication.

Thank you for this comment. As mentioned above, we have generated point mutants in the homodimerization interface, as well as chimeric NbNRC2-NbNRC3 proteins where residues at the homodimerization interface have been swapped. Several homodimerization mutants tested were autoactive or “trigger happy” (became active upon overexpression with Rx), suggesting that the biological implication of homodimerization is to contribute to immune receptor autoinhibition prior to activation. As for the swaps, we showed that the NB-LRR interprotomer interface (interface 1) is the main determinant for isolating different NRC paralogs, as swapping the residues at this interface from NRC3 into NRC2 abolished NRC2 self-association of NRC2.

4.    The authors shall consider revising what they consider as "resting" state as well as on the notion of the majority NRC2 form found in the plants being dimers.

Thank you for this comment. We define “resting” state as the conformation NbNRC2 adopts prior to activation by sensors. Following the submission of our manuscript to PLoS biology, a manuscript has been published in Nature where the authors report that the NRC2 homolog from tomato, SlNRC2, not only forms the same homodimers reported in our study but also forms tetramers as well as large filament-like assemblies. We have included a section in our paper covering this. Nonetheless, the observation that NbNRC2 mutants unable to form tetramers of filament-like assemblies are still able to form homohexamers suggests that these homodimers are the minimal activatable building block that these tetramers and filaments are made of, that is also capable of forming resistosomes and triggering cell death.

This can start with 1) clear indication of gel filtration data description with the strong notion of not detecting monomeric fractions;

This data is available in Figure S1, where we detected the main peak of NbNRC2 was a homodimer. We have now made this figure a main figure in order to make this data more clear.

2) indication of dimer configuration on the Figure 2 BN-PAGE gels, since the gels show two major forms (assuming the one around right below 480 marker should be dimer with the strong intensity, one can easily extrapolate that monomeric fraction might still exist as there is another band located below 242);

Thank you for this comment. As mentioned above, and in light of the recent paper published by Ma et al. on SlNRC2 which reports that NRC2 can form tetrameric assemblies, we now hypothesize that this band below the 480 marker corresponds to the tetrameric NbNRC2 assemblies, while the band around 242 corresponds to the homodimer. We do not exclude that a pool of NbNRC2 can be found as a monomer, but we believe that the majority of resting state NbNRC2 exists in either a homodimer or higher order (tetramer/filament) form. We have added a section covering this in the text.

3) discussion on another possibility of NRC2-dimer being an intermediate/primed state towards the assembly of full resistosome. The schematic model on the dimer splitting to monomers to reconfigure to a hexamer (or higher-order oligomer) is based on NRC4 structure and largely on that of Zar1. It is not very convincing how much contribution of the observed dimer making to the assembly of full resistosome as compared to the monomer-constituting resting state model. If the monomeric mutation is feasible to obtain, the biological meaning of having the dimeric resting state (either promotive or inhibitory) will be self-explaining.

Thank you for this comment. Do note that our schematic model is a working model, based on the data available. We have now included cell death assays with NbNRC2 homodimerization interface mutants, showing that some of these mutants become autoactive. This points towards the homodimer having an autoinhibitory role, and monomers being potentially autoactive. Our findings are also aligned with those of a recent publication, Ma et al. (2024) *Nature*, which reports analogous auto-inhibitory homodimeric and tetrameric assemblies for the tomato homolog of NRC2, SlNRC2. Moreover, we have also published a preprint reporting on the structure of the Rx-activated NbNRC2 oligomer, showing that it forms a hexameric assembly (Madhuprakash et al. (2024) *bioRxiv*). Nonetheless, the precise nature of the intermediate states that may arise during conversion of NbNRC2 from homodimer to homohexamer are not known. Whether monomeric intermediates appear or whether three homodimers trimerize leading to a hexameric resistosome remains to be tested. This speculation has been included in the discussion.

Minor comment 
It is a convention not to capitalize C in cryo-EM.

We have now modified the manuscript so that every instance of cryo-EM does not have a capitalized c.

------------------------------------


Reviewer #3: 

This straightforward manuscript by Selvaraj et al. describes the solution of the cryo-EM structures of dimer and hexamer of helper NLR NRC2. Similar to the other solved CNL resistosomes, NRC2 forms a ring, but with different number of subunits (6 instead of 5). The manuscript is well written and the conclusions are nicely supported by the biochemical data provided. Here are some points for the authors to consider for either discussion or experimental enhancement of their story. 

1.    In the model, the authors should include that with high ATP concentration, the NRC2 can form hexameric ring. Is this biological relevant? Have the authors tried to express NRC2 with high ATP concentration to see if HR reactions are changing in planta?

The formation of the hexameric ring upon high ATP concentrations is not biologically relevant, as the concentration of ATP that triggers this process are higher than the concentrations found in the cell. This assay was done mostly to identify suitable conditions to carry out NRC2 oligomerization assays in vitro. As such, we believe that infiltrating NRC2 with high ATP concentrations in the plant is not a biologically relevant experiment.

2.    Does the NRC2 hexameric ring have calcium channel activity as with ZAR1? Is the pore size large enough?

While we do not report on the structure of the hexameric NRC2 ring in this paper, we have recently reported on the structure of the activated NRC2 hexamer in a recent preprint by Madhuprakash et al. While we did not test the NRC2 hexamer for calcium channel activity, another recent preprint on the structure of the activated NRC4 hexamer by Liu et al. showed that it does exhibit calcium channel activity. Based on the fact that both the NRC2 and NRC4 hexamers are structurally similar and have similar pore sizes, we speculate that it should be capable of functioning as a calcium channel, similar to ZAR1. Because this paper is mostly focused on the resting state of NRC2 and on how homodimerization contributes to autoinhibition, not on the active hexameric form, we refrained from expanding on this beyond what is already in the discussion.

3.    The NRC2-GFP imaging data is intriguing. Any sign of condensate formation or phase separation, which may allow high local ATP concentration? How about the clear nuclear localization? Is it biological relevant (are the proteins inside the nuclei inactive dimer or activated hexamer)? If it is the activated form, are they associated with nuclear envelop for activation?

Our NRC2-GFP shows that NbNRC2 accumulates as filaments inside the cell, and that these filaments do not co-localize with NRC4. This data has now been validated structurally by Ma et al., who structurally characterized these NRC2 filaments using cryo-EM, and showed that they can assemble in vitro. The clear nuclear localization mentioned by the reviewer corresponds to the EV:GFP treatment, not NRC2:GFP. Free GFP is known to have nucleo-cytoplasmic localisation. We do not observe nor report any nuclear localization for NRC2 in this or other publications from our groups (see also Duggan et al. 2021, PNAS). As for the activated form of NRCs, we previously showed that activated NRC2 and NRC4 accumulate at the plasma membrane, forming punctate structures (see Duggan et al. 2021, PNAS and Contreras et al. 2023, EMBOJ and Sci. Adv). Nonetheless, this is outside of the scope of this paper so we have refrained from delving deeper into this in this manuscript.

4.    It is unclear how the Rx NB activates the hexamer formation. Is this specific to certain Rx residues?

A recent study by Huang et al. 2023 (https://www.biorxiv.org/content/10.1101/2023.12.12.571219v1.full.pdf , cited in the paper) suggests that activation of NRC helpers by sensors such as Rx occurs via transient interactions, as shown using proximity labelling. In another study by our group, we showed that the NB domain of Rx and other sensors is sufficient to trigger NRC2 hexamer assembly (Contreras, Pai et al. 2024, New Phytologist), and have extensively discussed our hypotheses on how the RxNB activates hexamer formation in this study. Our model is that NB domain of sensors transiently interact with helpers to trigger hexamerization via transient sensor-helper interactions. However, the exact interface via which Rx NB interacts with NRC2 is not known yet. We are currently working on identifying said interface and understanding the specific residues in the NB domain of Rx and other sensors that are responsible for NRC activation. While this is highly exciting, the specifics of RxNB-NRC communications are not the focus of this study and have been covered extensively in our other publication. Moreover, we feel that understanding in more detail the specific amino acids involved is beyond the scope of this paper.
